# The Impact of Visualization Format and Navigational Options on Laypeople’s Perception and Preference of Surgery Information Videos: Randomized Controlled Trial and Online Survey

**DOI:** 10.2196/12338

**Published:** 2018-11-22

**Authors:** Marie Eggeling, Martina Bientzle, Thomas Shiozawa, Ulrike Cress, Joachim Kimmerle

**Affiliations:** 1 Knowledge Construction Lab Leibniz-Institut fuer Wissensmedien Tuebingen Germany; 2 Institute of Clinical Anatomy and Cell Analysis Eberhard Karls University Tuebingen Tuebingen Germany; 3 Department of Psychology Eberhard Karls University Tuebingen Tuebingen Germany

**Keywords:** attitude, decision aids, emotions, informed decision making, knowledge acquisition, medical decision making, surgery, video

## Abstract

**Background:**

Patients need to be educated about possible treatment choices in order to make informed medical decisions. As most patients are medical laypeople, they find it difficult to understand complex medical information sufficiently to feel confident about a decision. Multimedia interventions such as videos are increasingly used to supplement personal consultations with medical professionals. Former research has shown that such interventions may have a positive effect on understanding, decision making, and emotional reactions. However, it is thus far unclear how different features of videos influence these outcomes.

**Objective:**

We aimed to examine the impact of visualization formats and basic navigational options in medical information videos about cruciate ligament surgery on recipients’ knowledge gain, emotions, attitude, and hypothetical decision-making ability.

**Methods:**

In a between-group randomized experiment (Study 1), 151 participants watched 1 of 4 videos (schematic vs realistic visualization; available vs unavailable navigational options). In a separate online survey (Study 2), 110 participants indicated their preference for a video design. All participants were medical laypeople without personal experience with a cruciate ligament rupture and were presented with a fictional decision situation.

**Results:**

In Study 1, participants who used navigational options (n=36) gained significantly more factual knowledge (*P*=.005) and procedural knowledge (*P*<.001) than participants who did not have or use navigational options (n=115). A realistic visualization induced more fear (*P*=.001) and disgust (*P*<.001) than a schematic video. Attitude toward the surgery (*P*=.02) and certainty regarding the decision for or against surgery (*P*<.001) were significantly more positive after watching the video than before watching the video. Participants who watched a schematic video rated the video significantly higher than that by participants who watched a realistic video (*P*<.001). There were no significant group differences with regard to hypothetical decision making and attitude toward the intervention. In addition, we did not identify any influence of the visualization format on knowledge acquisition. In Study 2, 58 of 110 participants (52.7%) indicated that they would prefer a schematic visualization, 26 (23.6%) preferred a realistic visualization, 17 (15.5%) wanted either visualization, and 9 (8.2%) did not want to watch a video at all. Of the participants who wanted to watch a video, 91 (90.1%) preferred to have navigational options, 3 (3.0%) preferred not to have navigational options, and 7 (6.9%) did not mind the options.

**Conclusion:**

Our study indicates that the perception of medical information videos is influenced by their design. Schematic videos with navigational options are the most helpful among all videos to avoid negative emotions and support knowledge acquisition when informing patients about an intervention. The visualization format and navigational options are important features that should be considered when designing medical videos for patient education.

**Trial Registration:**

Deutsches Register Klinischer Studien DRKS00016003; https://www.drks.de/drks_web/ navigate.do?navigationId= trial.HTML&TRIAL_ID=DRKS00016003 (Archived by WebCite at http://www.webcitation.org/746ASSAhN)

Original Paper

## Introduction

### Background

In clinical practice, doctors and patients are sometimes faced with a situation that has no clearly optimal treatment. Nonetheless, they have to make a decision on the most-appropriate option. Such decisions are *preference sensitive*, because they should consider individual circumstances and preferences of the patient as well as scientific evidence. Most patients prefer making shared or autonomous medical decisions [1]. Although informed consent is required for any medical treatment and shared decision making has become more common in the last few decades, many patients find it difficult to participate in the decision-making process [2]. To make an informed decision, patients have to acquire knowledge about the process, risks, benefits, and alternatives. The decision-making process is also influenced by patients’ attitudes, and patients should be able to make a choice that reflects their personal values and preferences [3]. Several studies have shown that shared decision making has a positive impact: Patients have more realistic expectations, feel more satisfied with the decision, and show more treatment cooperation [4]. In addition, they make more conservative treatment decisions: A previous review showed that the number of people undergoing major elective surgery decreased [5] as compared to usual care when the participants actively participated in shared decision making, and many patients chose a less invasive option [6] when they watched an educational video in addition to the standard encounter with a physician.

Implementing shared decision making is challenging. Many patients feel that they lack the knowledge to make an informed decision and therefore underestimate the importance of personal preferences and individual experiences [7,8]. Patients often remember little of the verbal information they received during a consultation, and doctors tend to overestimate patients’ level of comprehension [9,10].

### Decision-Support Tools

Decision-support tools are increasingly used to support patients who have to make a medical decision. They can be applied before, during, or after a clinical encounter; provide evidence-based information about options, risks, and benefits; and support the decision-making process by helping patients imagine the different options and clarify their personal values [11]. Decision-support tools are often computer based and include different media formats such as texts, pictures, and videos. They are not intended to substitute a personal consultation with an experienced doctor, but to prepare patients for this consultation or allow them to learn more about the subject, revisit information, and visualize complex processes or structures [12,13].

Experimental studies that explicitly tested the use of decision aids during the informed-consent process found that the aids consistently had a positive impact on knowledge gain [13-18]. Only a few studies found a significantly higher satisfaction with the process [13,14,17], and usually, no difference in anxiety was observed [13,14,18,19]. However, these studies used different tools, ranging from complex multimedia interventions to short videos or pamphlets. In addition, they examined diverse medical fields with various kinds of decisions. Most importantly, only a few studies investigated the decision-making process, because the decision itself was often already made.

A review by Stacey and colleagues [5] showed that in 105 studies that compared decision aids with usual care, decision aids improved patients’ knowledge about their options and reduced the decisional conflicts stemming from feeling uninformed and unclear about their personal values. Furthermore, decision aids stimulated people to take a more active role in decision making and increased the accuracy of their risk perceptions. In another review, Wilson and colleagues [20] compared multimedia and print health materials and found that the former were superior to print, but there were no significant differences in more than half of the outcome measures. Notably, the interventions and outcomes in these studies were diverse. Multimedia tools have the potential to support patient education and decision making better than a verbal consultation or print materials alone, but there is a need to investigate the circumstances under which they have a positive impact on knowledge and decision making.

### Videos to Support Informed Decision Making

Videos are often included in many decision-support tools, usually as part of a more complex multimedia intervention. The use of videos as a source of medical information has increased in the past years [21] in a professional context as well as on platforms like YouTube [22], indicating that laypeople are interested in this format. Videos might help impart medical information to laypeople, because they can make complex anatomical information more vivid than text or pictures alone and provide patients a visual impression of a particular treatment. Additionally, patients can watch the videos more than once, and videos are a cost-effective way to communicate information [23]. Thus, videos may be a good resource for supporting informed decision making and appropriate for use in decision-support tools. Several studies have found that watching an informational video in addition to having access to classical information sources (consultation and information brochure) had a positive effect on knowledge gain [17,24-27]. Regarding emotions, there have been different findings: Pager [25] found that patients who had watched a video explaining the expectations from cataract surgery expected the surgery to be riskier and more unpleasant, but felt less anxious during the surgery and were more satisfied after the surgery. A study with patients who underwent coronary artery bypass surgery showed that watching a video resulted in less fear and a stronger feeling of subjective well-being before the surgery and less depression after the surgery [28]. A few other studies showed a reduction of fear after watching a video [29-31], whereas others did not show such an effect [18,19]. Regarding decision making, Volandes and colleagues [32] found that watching a video had a positive impact on the ability to make a decision. In a study by Chiou and Chung [33], a video intervention reduced both uncertainty and decisional regret.

In summary, research suggests that videos can be useful in improving patient education, may regulate emotions, and have the potential to support decision making. However, the videos used in these studies were diverse, which may explain the difference in some aspects of previous findings. Thus far, there has been no research on the potential impact of different designs of video formats. In addition, only a few studies have specifically investigated the influence of different videos on decision making. For patient counseling, it is important to examine which features of educational videos may promote knowledge gain, evoke or avoid negative emotions, and support attitude formation in medical decision making.

### Design of Medical Information Videos

The design of educational videos can differ in many ways. One possible variation for the *visualization format* is the use of schematic or realistic presentations. A schematic video consists of animated line drawings that present the most-relevant parts or components but omit negligible details. In contrast, a realistic video shows real representations and processes and depicts them in their actual complexity. In the case of a surgery video, a realistic film would show an actual surgery on a real body. Studies in nonmedical contexts have shown that schematic videos led to better learning outcomes [34,35] and were easier to understand than realistic videos, especially when people had little prior knowledge [36]. However, knowledge gain is not the only important outcome of educating patients. When designing a video to support informed, value-concordant decision-making, one needs to bear in mind that patients also need to form an attitude toward the intervention of their choice. As realistic videos are more vivid than schematic ones, they have the potential to generate more negative emotions, in particular, fear and disgust. However, a realistic depiction might make the actual procedure of an intervention easier to imagine, which could reassure patients of the realities of the potential surgery and support the formation of an attitude.

Another type of variation in educational videos is the amount of *user control*. The availability of basic navigational options to stop and skip forward or backward was found to facilitate the learning process in nonmedical domains [37-39]. To our knowledge, no studies have thus far investigated the effect of such navigational options on emotions, attitudes, or decision making in the medical context.

Herein, we conducted two studies to compare differently designed videos in a randomized controlled experiment and to examine people’s preferences for particular designs of medical information videos in an online survey.

### Hypothesis and Research Questions

In our first study, we aimed to compare different visualization formats (schematic vs realistic visualization) and basic navigational options (available or unavailable) in medical information videos in terms of their impact on knowledge, emotions, and attitudes. In addition, we examined the influence of the two abovementioned factors on patients’ decision to undergo one of two medical treatments. As a video topic, we chose *arthroscopic cruciate ligament surgery*, which is a common orthopedic surgical procedure and a frequently performed treatment after a cruciate ligament rupture [40,41]. Another frequently used, alternative treatment is intense physiotherapy. We decided on this topic because studies comparing the two methods are unclear about whether surgery or physiotherapy is the clearly superior treatment [42-44]. Therefore, each patient would have to make an individual decision about undergoing this surgery. In addition, a cruciate ligament rupture is a frequent sports accident and therefore easy to imagine for a sample of healthy participants.

As the influence of video design on attitude formation and decision making in a medical context has not been examined thus far, we investigated these influences in the form of explorative questions. Additionally, we asked participants for a general evaluation of the video.

Previous research has found that schematic videos made learning of complex topics easier as compared to realistic videos [34-36]. Accordingly, we expected higher levels of factual knowledge and procedural knowledge with a schematic video (H1). As the availability of basic navigational options had a positive impact on knowledge gain in other studies [37-39], we expected higher factual knowledge and procedural knowledge with the availability of basic navigational options (H2). Laypeople are not accustomed to watching a surgical procedure on a human; therefore, it might evoke negative emotions. Thus, we assumed that participants who watched a realistic operation would experience more fear and disgust than those who watched a schematic video (H3). Finally, we predicted a significant increase in certainty of the decision after watching the video, as the additional information should support the decision-making process (H4).

In our second study, we wanted to learn more about people’s preferences for visualization formats and basic navigational options to complement the results of the first study. As in Study 1, we used the topic *arthroscopic cruciate ligament surgery*. In contrast to Study 1, which was conducted as a laboratory experiment, Study 2 was performed as an online survey.

## Methods

### Ethical Approval

This research was performed in accordance with the Declaration of Helsinki and received full approval by the ethics committee of the Leibniz-Institut fuer Wissensmedien (approval number: LEK 2017/041).

The trial was not preregistered as Study 1 was conducted before trial registration became standard policy at the Leibniz-Institut für Wissensmedien.

### Study 1

#### Study Setting

We conducted a randomized controlled experiment in a laboratory setting. The participants were placed in a hypothetical situation where they would have to confront the possibility of surgery. They watched an educational video and responded to several questionnaires about their knowledge (factual and procedural knowledge), emotions (fear and disgust), and attitude toward the intervention as well as their decision and certainty of the decision.

#### Participants

**Figure 1:**
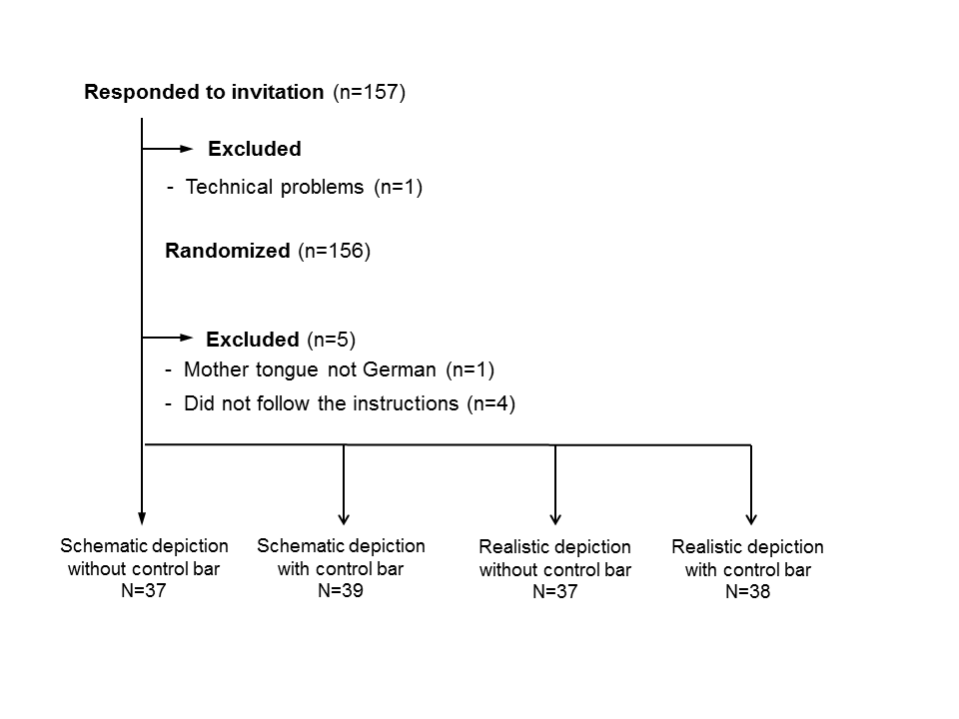
Sampling procedure of Study 1.

To be able to detect a medium effect size (ρ=.30, α=0.05, 1-β=0.95), a sample size of 40 per condition was required. The experiment was performed with 157 laypeople who were randomly recruited from the participant database of the Leibniz-Institut fuer Wissensmedien and invited via email. Registration in this database was voluntary and open to everyone who was willing to participate in empirical studies. Medical or sports students and people working in a medical profession were not invited to participate, because we aimed to include participants who had no or little prior professional knowledge about the topic and wanted to determine how laypeople, in particular, reacted to the videos. In the invitation, potential participants were informed that during the experiment, they might watch a video demonstrating the surgery on a body donor. They were repeatedly informed that they could stop the video at any point without any negative consequences. Of the 157 participants who were initially recruited, 6 participants were excluded: 1 participant had technical problems, 1 had specified a mother tongue other than German, and 4 did not follow the instructions (ie, did not read the information sheet or consent to have their computer screen recorded while the video was running; see Procedure section). The remaining 151 participants (mean 25.90 years, age range 19-67 years, Table 1) were randomly assigned to the 4 experimental conditions. The sampling procedure is shown in Figure 1. Across the 4 experimental conditions, the participants did not differ in age (F(3,147)=1.62; *P=*.19; n=151), distribution of gender (χ²=7.44; *P*=.28; n=151), occupation (χ²=7.40; *P*=.83; n=151), or education (χ²=4.41; *P*=.62; n=151). All participants provided written informed consent. The study lasted for approximately 45 minutes and was compensated with 6 Euros.

**Table 1:** Demographic characteristics of the participants in Study 1.

Characteristics		n (%)
**Age (years)**		
	19-24	78 (51.7)
	25-30	57 (37.7)
	≥31	16 (10.6)
**Gender**		
	Female	105 (69.5)
	Male	45 (29.8)
	Other	1 (0.7)
**Occupation**		
	University student	132 (87.4)
	Employee	10 (6.6)
	Other	9 (6.0)
**Education**		
	No graduation	1 (0.7)
	School-leaving certificate	87 (57.6)
	University degree	63 (41.7)

#### Procedure

**Figure 2:**
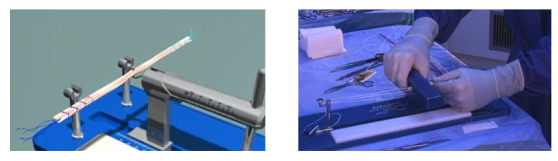
Schematic (left) and realistic (right) video formats.

All the instructions and questionnaires were presented on a computer screen. After reading the study description and signing the informed consent form, participants answered questions about their demographic data (age, gender, occupation, and education). Subsequently, they were asked to place themselves in a fictional situation where they had suffered a cruciate ligament rupture in a sports accident and were faced with a choice for or against surgery. They were asked about their decision (how likely they were to hypothetically undergo surgery), certainty of this decision, and attitude toward the potential surgery (premeasures). Thereafter, they were asked to imagine that before the next consultation, their doctor gave them an information brochure about their injury and recommended a video on the possible surgery. At this point, the same information sheet was handed out to every participant. It included general information on the location and function of the cruciate ligament and consequences of an injury and treatment options (surgery or intense physiotherapy). This information was provided in a neutral manner. Patients were also informed that in medical research, thus far, no treatment was found to be clearly better than the others and that each patient had to make an individual decision. This information sheet comprised 679 words. After they finished reading this sheet, the participants watched one of four different videos, depending on their experimental condition. They were randomly assigned to the conditions by the survey program Qualtrics (Qualtrics LCC, Provo, Utah). The videos differed in their visualization format (realistic vs schematic depiction; Figure 2) and the availability of basic navigational options (with or without a control bar to stop and skip forward or backward). They were similar in terms of content (focusing on the surgical procedure). The realistic and schematic videos showed the same procedure in the form of a real operation on a human body involving real people or in the form of an animated line drawing. Both videos had identical spoken text and the same duration (approximately 3.5 minutes; Multimedia Appendix 1). The contents of the information sheet and the videos were reviewed by physicians and professional anatomy educators. All participants were allowed to take notes while watching the video. We recorded the computer screen while the video was playing in order to examine how participants used the navigational options. When the participants had finished watching the video, their notes and the information sheets were collected. Subsequently, they answered questions about their decision, certainty of the decision, and attitude toward the potential surgery (postmeasures) again. In addition, they answered questions about their emotions while watching the video and their general evaluation of the video. Finally, they received a knowledge test that consisted of questions about the information presented in the video.

#### Measures

The knowledge test consisted of 6 items. Five items covered *factual knowledge* about the information provided in the video. The questions were either multiple choice or required the input of a single word or number. The mean score of these items was calculated, resulting in a maximum score of 1 point. *Procedural knowledge* was evaluated using a sorting task, where participants had to put 5 operation steps in the right order. One point was awarded to each correctly ordered step; this score was divided by 5, again resulting in a maximum score of 1 point.

To measure the participants’ emotions while watching the video, we used the subscales *fear* and *disgust*, with 3 items each from the modified Differential Affect Scale [45]. On a 5-point Likert scale, participants indicated how strongly they felt this emotion during the video presentation. The subscales showed good or excellent internal consistencies (Cronbach *α*_disgust_=.95, Cronbach *α*_fear_=.82). *Attitude* toward the intervention was measured using 4 bipolar items on a 7-point scale before and after watching the video [3]. Internal consistencies were acceptable or good (Cronbach *α*_att-pre_=.70, Cronbach *α*_att-post_=.80). The participants’ hypothetical *decision* was noted before and after watching the video. On a 5-point scale, they indicated how likely they were to hypothetically undergo surgery in the situation they were given.

To measure *certainty* regarding the decision, participants were asked in 5 items how certain they felt about their decision for or against the surgery. All responses were given on a 5-point scale, and the items showed acceptable internal consistencies (Cronbach *α*_cert-pre_=.66, Cronbach *α*_cert-post_=.67). General *evaluation* of the video was measured using 4 items. Internal consistency was acceptable (Cronbach *α*_sat_=.66). Multimedia Appendix 2 provides an overview of all measures.

#### Analysis

Data analysis was performed using IBM SPSS 22 statistics for Windows (International Business Machines Corporation, Armonk, New York). To test for differences between the conditions, we performed analyses of variance, including repeated measure analysis for the pre-post comparisons. We report all data as means and standard deviations. The level of significance was set at *P*<.05. The partial eta-squared value was calculated as effect sizes of mean differences.

### Study 2

#### Participants

The online survey was performed with 110 laypeople (mean 24.05 years, age range 18-72 years, Table 2) who were recruited from the same participant database as Study 1 and invited via email. Medical or sports students, people working in a medical profession, and persons who had already participated in Study 1 were not invited to participate in this study. Participants had the option to participate in a raffle to win vouchers for an online shop. The survey lasted for approximately 5-10 minutes.

#### Procedure

The online survey could be completed on any electronical device. Participants read the study description, provided written informed consent, and answered questions regarding their demographic data. Subsequently, they were given the same situation as in Study 1 and answered 2 questions regarding the video they would choose to watch in this situation. To show the participants what the schematic and realistic videos would look like, they were presented with still images from the 2 videos (Figure 2).

#### Measures

In the first question, participants were asked if they would like to watch (a) a schematic video, (b) a realistic video, (c) any video without preference for the visualization format, or (d) no video at all. If they answered a, b, or c, they were asked if they would like to have basic navigational options (yes, no, I don’t care). The survey only consisted of these basic questions and did not include any manipulation, since this study was intended to identify people’s general preferences for medical videos.

#### Analysis

Chi-squared tests were used to test for differences in frequencies.

**Table 2:** Demographic characteristics of the participants in Study 2.

Characteristics		n (%)
**Age (years)**		
	18-24	76 (69.1)
	25-30	26 (23.6)
	≥31	8 (7.3)
**Gender**		
	Female	85 (77.3)
	Male	24 (21.8)
	Other	1 (0.9)
**Occupation**		
	University student	98 (89.1)
	Employee	10 (9.1)
	Other	2 (1.8)
**Education**		
	No graduation	4 (3.6)
	School-leaving certificate	83 (75.5)
	University degree	23 (20.9)

## Results

### Study 1

In contrast to Hypothesis 1, there were no significant group effects of the visualization format regarding *factual knowledge* (schematic: mean 0.62, SD 0.23; realistic: mean 0.60, SD 0.22; *P*=.32) or *procedural knowledge* (schematic: mean 0.77, SD 0.26; realistic: mean 0.82, SD 0.29; *P*=.57).

As per Hypothesis 2, we expected participants who were given basic navigational options to gain more factual knowledge and procedural knowledge than participants without navigational options. However, we did not observe the expected group differences (factual knowledge: *P*=.10, procedural knowledge: *P*=.51). There was a significant difference between the participants who had used the navigational options at least once (n=36) and those who had not used the options at all (n=115). Participants who used the options at least once performed significantly better in both *factual knowledge* (navigation used: mean 0.70, SD 0.22; navigation not used: mean 0.58, SD 0.22;_*part.*_*η*^*2*^=.05, F(1,147)=8.18, *P*=.005), and *procedural knowledge* (navigation used: mean 0.93, SD 0.17; navigation not used: mean 0.75, SD 0.31;_*part.*_*η*^*2*^=.08, F(1,147)=11.94, *P*<.001).

In support of Hypothesis 3, participants who had watched the realistic video experienced more *fear* (schematic: mean 1.49, SD 0.68; realistic: mean 1.96, SD 1.04;_*part.*_*η*^*2*^=.07, F(1,147)=11.12, *P*=.001) and *disgust* (schematic: mean 1.47, SD 0.79; realistic: mean 2.48, SD 1.28;_*part.*_*η*^*2*^=.19, F(1,147)=34.14, *P*<.001) than participants who had watched the schematic video.

In support of Hypothesis 4, certainty about the decision was significantly higher after the video presentation (mean 2.64, SD 0.68) than before the presentation (mean 2.22, SD 0.66) (_*part.*_*η*^*2*^=.34, F(1,147)=75.71, *P*<.001). In addition, we found no differences between the video types.

Regarding our explorative analysis, we found no significant group differences for attitude toward the intervention (all *P*>.09). However, there was a significant pre-post effect on attitude: Participants’ attitude toward the intervention was more positive after watching the video (mean 4.31, SD 1.07) than before watching the video (mean 4.16, SD 0.91) (_*part.*_*η*^*2*^=.35, F(1,147)=5.27, *P*=.02).

There were no significant group effects on decision (all *P*>.095) or certainty about the decision (all *P*>.54). However, we found that participants who had watched a schematic video rated this video significantly higher (mean 4.36, SD 0.49) than that by participants who had watched a realistic video (mean 3.86, SD 0.86) (_*part.*_*η*^*2*^=.12, F(1,147)=20.34, *P*<.001).

### Study 2

Of the 110 participants, 101 (91.8%) wanted to watch a video and 9 (8.2%) preferred not to watch any video (*χ*^*2*^(1, 110)=76.95, *P*<.001). Regarding the visualization format, 58 participants (52.7%) preferred to watch a schematic video, 26 (23.6%) opted for a realistic video, and 17 (15.5%) did not favor any format (*χ*^*2*^(3, 110)=50.36, *P*<.001). Of those who wanted to watch a video, 91 participants (90.1%) preferred to have basic navigational options, 3 (3.0%) preferred not to have navigational options, and 7 (6.9%) did not care (*χ*^*2*^(2, 101)=146.69, *P*<.001).

## Discussion

### Study 1

The results of this experiment showed that the use of navigational options supported the development of factual and procedural knowledge. Watching a schematic video led to fewer negative emotions, and the participants liked the schematic video better than the realistic video format. However, the participants were randomly assigned to watch one visualization format and did not have the option to select their preferred format or watch no video at all. Therefore, it was important to address the extent to which people would be interested in watching an information video about surgery and the video design they would prefer. To this end, we conducted an online survey to expand on the findings of the experimental study.

### Study 2

The results of the survey showed that most participants were generally interested in medical information videos, which is in line with former research [46]. Furthermore, schematic videos were preferred over realistic videos, and most participants indicated that they would like to have navigational options. These findings support the results of the experimental study, where a schematic visualization caused fewer negative emotions and was better than a realistic visualization and where the use of navigational options led to better recall of information covered in the video.

### General Discussion

The research presented here aimed to investigate the potential of medical information videos for patient education and decision making. Such videos may be used in preparation for or follow-up of a medical consultation to support decision making. Former research has reported that such tools may support knowledge acquisition [5], but their benefit for decision making is unclear. Moreover, the results of their impact on emotions are varied. One problem was the diversity of videos used in previous studies, due to which it was difficult to identify the reasons for different findings. In our first study, we aimed to examine how visualization of differently designed information videos influenced learning, emotions, attitude, and decision making.

The laboratory experiment showed that watching a video about cruciate ligament surgery modified participants’ attitude toward the intervention and increased their certainty about the hypothetical decision for or against surgery. These results are in line with those of other studies that reported positive effects of medical videos when informing viewers about an upcoming surgical procedure (eg, [15,47,48]). People require support to make informed decisions and reassurance to make individual choices for treatments that are consistent with their own personal values; our results suggest that videos may be a suitable medium to facilitate this process. The video format did not have a differential impact on the certainty of the decision, indicating that all videos were equally helpful in decision making.

The use of basic navigational options resulted in better performance on a knowledge test. In contrast to the findings of Hasler and colleagues [39], we found no benefit of the navigational options when they were made available but not used. Since participants who used the navigational options automatically spent more time with the video, their better performance in the knowledge test could have resulted from longer exposure to the content. This finding could also reflect differences in motivation among people who used or did not use the navigation tool. Nevertheless, this result is interesting, as it implies that watching a video only once may not be enough and repetitions and pauses are beneficial to process the content. This finding is in agreement with that of Wilson and colleagues [20], who reported that participants who reviewed an information brochure at home performed better than participants who did not review the brochure at home. One advantage of multimedia tools as compared to personal consultation is that learning may be adjusted to the viewers’ own speed and preferences, which is beneficial for learning.

In our experimental study, a schematic visualization was associated with fewer negative emotions and a more positive evaluation than a realistic visualization. This finding is particularly interesting, because recent technological developments have increasingly relied on schematic representations of surgical interventions (eg, [49]). In addition, the majority of participants in the additional online survey stated that they would prefer to watch a schematic video with navigational options, indicating that the design of medical information videos about surgical interventions can affect the participants’ perception (eg, [50]) and might explain why studies have found different results for the impact of interventions on, for example, emotions.

The studies presented here have some limitations. First, the participants’ decision-making process was hypothetical, as our participants were not patients. Although this approach allowed us to perform the study in a controlled experimental setting and choose a situation that might be relatively easy to imagine, the motivation of our participants to engage in the situation may be lower than that among patients, for whom the situation would be personal and relevant. Patients may place importance on other aspects and react differently to the videos; in addition, some of our participants may have faced difficultly in imagining their feelings and thoughts in the described situation. Second, a large number of our participants were university students and therefore relatively young and well educated. Consequently, one should exercise caution when generalizing these findings to an entire population. For example, younger people may be physically more active and may therefore be more likely to opt for surgery than older people, which is supported by the fact that the average attitude toward surgery was positive. Some previous studies found that patients with low educational levels benefited significantly more from multimedia interventions than patients with high educational levels [12,15,16]. As such, our videos may have been more useful for people with a low educational background. In addition, educational videos may need to be designed differently depending on the target audience. Third, our results only indicate that watching the videos supported participants’ decision-making process, but did not explain the manner or reason for this finding. Future studies could resolve this limitation by asking participants to verbally explain their decision. Although we asked participants for prior experiences with the subject, we did not test their knowledge prior to the intervention. However, as the participants were randomly assigned to the experimental groups, this omission may not be a problem. Nonetheless, inclusion of a pretest would allow evaluation of learning development and consideration of individual differences.

We informed participants that there were two possible treatment methods, but showed them a video that only addressed one method—surgery—because we wanted to focus on one intervention and clarify the reason for the possible effects observed. The disadvantage of this approach was that participants learned more about the surgery than about the alternative treatment, which likely influenced their attitude and decision-making process. Future studies with a larger sample size should create different videos about the different treatment options and compare their effects on the outcome variables. In addition, they should aim to transfer the research questions into more-realistic settings to determine if patients can benefit from such videos in the same way as the participants in our experiment did and show similar preferences regarding the video design.

### Practice Implications

Medical educational videos are useful for providing knowledge to laypeople with little prior experience and support informed decision making. Our studies showed that the visualization format and user control options should be considered in the design of such videos. Our findings suggest that schematic videos with navigational options, along with encouragement to use them, may be most helpful in avoiding negative emotions and supporting knowledge acquisition.

### Conclusions

Videos are a good medium for educating patients about medical topics and should be used as decision-support tools to make complex information more vivid and easier to understand for laypeople. Our studies show that the design of such videos can influence information processing. The schematic visualization caused fewer negative emotions, was liked better than the realistic visualization, and was preferred by more than half of the participants in our survey. In contrast, almost one-fourth of the participants showed interest in a realistic presentation format. To increase satisfaction and personal benefit, different types of visualizations should be offered and patients should be given the opportunity to decide individually which type they prefer. This approach would be easy to realize in decision-support tools. Since participants spent more time with the video and acquired more knowledge with the use of navigational options, navigational options should be made available to participants and participants should be encouraged to actively use them, for instance, to pause and repeat difficult or interesting parts of the video.

## Appendix

Multimedia Appendix 1 Script of the audio track in the realistic and schematic video formats.

Multimedia Appendix 2 Measures in Study 1.

Multimedia Appendix 3 CONSORT‐EHEALTH checklist (V 1.6.1).
