# Supplementary material for: The Impact of Visualization Format and Navigational Options on Laypeople’s Perception and Preference of Surgery Information Videos: Randomized Controlled Trial and Online Survey
Source: J Particip Med. 2018 Nov 22;10(4):e12338. doi: 10.2196/12338 (PMC7434097; doi:10.2196/12338)
Supplement: Multimedia Appendix 1 [file jopm_v10i4e12338_app1.pdf]

Multimedia Appendix 1

Script of the audio track in both video versions

At the beginning of the operation, the tendon, which will later be used to create the transplant, is removed. The surgeon makes a short incision on the inside of the shin where the tendon is located. A thread is wrapped around the semitendinosus tendon to separate it from the surrounding structures. Then the tendon tissue is disconnected from the bone. The loop is used to insert a ring knife. Using the knife, the tendon is completely separated from the muscle tissue and removed.

The extracted semitendinosus tendon is now processed into a transplant. Then the transplant is measured and stretched in a device referred to as a transplant master to obtain the necessary basic tension before it is inserted into the knee.

Next, drill channels are created in the knee in which the transplant will later be inserted. Two channels are drilled: one at the front of the shin and the second in the area of the thighbone. The diameter of the drill channels is determined by the thickness of the transplant. It is important that they have exactly the right diameter so that the transplant can later grow in optimally. The channels are first drilled with a small diameter and then enlarged. As a result, small corrections are still possible. Later the transplant will be pulled through using what is referred to as a shuttle thread. Using a guidewire, the shuttle thread is pulled into the drill holes and secured there.

The transplant is hooked into the shuttle thread to pull it into the joint. At the upper end of the thread is a button, which folds down when it has been completely pulled through. In this way, it firmly anchors the transplant on the bone surface.

Now the transplant is pulled into the bone channels. An arthroscope allows the transplant to be seen all the time on a screen in the operating room.

After the transplant has been pulled through, it is important to retighten it vigorously. This should happen in different positions so that the knee will have good tension later in any position. To achieve this, the knee is stretched and flexed several times. Subsequently, the other end of the transplant is fixed with a special button. These buttons are bioabsorbable, meaning that they dissolve within 1-2 years.

Once the transplant is adequately fixed, the wounds are closed and sewn back together and the surgery is completed.
