# Supplementary material for: The Impact of Visualization Format and Navigational Options on Laypeople’s Perception and Preference of Surgery Information Videos: Randomized Controlled Trial and Online Survey
Source: J Particip Med. 2018 Nov 22;10(4):e12338. doi: 10.2196/12338 (PMC7434097; doi:10.2196/12338)
Supplement: Multimedia Appendix 2 [file jopm_v10i4e12338_app2.pdf]

Multimedia Appendix 2

Measures Study 1

Knowledge

Factual knowledge

- Which instruments were used to remove the tendon?
- Which tendon was used to make the graft for the anterior cruciate ligament?
- How many drill holes have been created?
- Where were the channels drilled in which the graft was implanted?
- What is the name of the instrument in which the graft was clamped?

Procedural knowledge

Sorting task, where participants had to put five operation steps in the right order:

- Removal of the tendon
- Grafting
- Bore of bone channels
- Stretching and bending the knee
- Sewing of the wound

Emotions

Fear

- Anxious
- Fearful
- Frightened

Disgust

- Disgusted
- Repulsed
- Revolted

Attitude

For me, surgery after a rupture of the anterior cruciate ligament would be ...

- beneficial – harmful
- important – unimportant
- a bad thing – a good thing
- unpleasant – pleasant

Decision

Would you choose to have surgery for a ruptured anterior cruciate ligament in the described situation?

Certainty regarding decision

- How certain are you regarding your decision for or against surgery?
- Based on my current knowledge I believe that I can make a decision for or against surgery.
- I would like further information about the topic before I can make a decision for or against surgery.
- I would like to consult a capable and recognized expert in this field and rely on her/his judgement regarding the decision for or against surgery.
- I would definitely obtain a second opinion before I decide for or against surgery.

Video evaluation

- How did you like the video in general?
- How well did the video impart the relevant knowledge about a cruciate ligament surgery?
- As an affected patient, how much would you like to watch such a video to inform yourself about a cruciate ligament surgery?
- How useful do you consider the video for patients who inform themselves about a cruciate ligament surgery?
